# Use of FGF-23 and sαKlotho for Risk Stratification in Patients with Acute Heart Failure

**DOI:** 10.3390/jcm14030860

**Published:** 2025-01-28

**Authors:** Joanna Płonka, Agnieszka Olejnik, Anna Klus, Ewa Gawrylak-Dryja, Natalia Wężyk, Lidia Rzepiela, Klaudia Dąbrowska, Krzysztof Nalewajko, Tomasz Porażko, Iwona Bil-Lula, Marek Gierlotka

**Affiliations:** 1 Department of Cardiology, University Hospital, Institute of Medical Sciences, University of Opole, 45-401 Opole, Poland; natalia_wezyk@o2.pl (N.W.); lrzepiela@wp.pl (L.R.); klaudia.da@gmail.com (K.D.); krzysztof.nalewajko@uni.opole.pl (K.N.); marek.gierlotka@uni.opole.pl (M.G.); 2 Department of Medical Laboratory Diagnostics, Division of Clinical Chemistry and Laboratory Haematology, Faculty of Pharmacy, Wrocław Medical University, 50-556 Wrocław, Poland; agnieszka.olejnik@umw.edu.pl (A.O.); iwona.bil-lula@umw.edu.pl (I.B.-L.); 3 Department of Clinical Biochemistry and Laboratory Diagnostics, Institute of Medical Sciences, University of Opole, 45-401 Opole, Poland; klusanna@interia.pl (A.K.); ewa.gawrylak-dryja@usk.opole.pl (E.G.-D.); 4 Department of Internal Medicine and Nephrology, Institute of Medical Sciences, University Hospital in Opole, 45-401 Opole, Poland; tporazko@uni.opole.pl

**Keywords:** acute heart failure, biomarker, fibroblast growth factor 23, prognostication, soluble αKlotho

## Abstract

**Background/Objectives:** Soluble αKlotho (sαKlotho) and fibroblast growth factor 23 (FGF-23) are increased in acute heart failure (AHF). This study aimed to assess changes in serum sαKlotho and FGF-23 concentrations during an episode of AHF as well as the usefulness of both biomarkers for predicting long-term prognosis. **Methods:** The study included 104 consecutive patients hospitalized in t he intensive cardiac care unit due to AHF (mean age, 65.8 ± 14.6 years; mean ejection fraction, 31.4% ± 14). New-onset AHF was reported in 43.3% of the population. Blood samples were measured at entry and on discharge from hospital. The main clinical outcomes assessed in this study were all-cause mortality or rehospitalization due to HF during a 3-year follow-up. **Results:** At admission sαKlotho, FGF-23, and NT-pro BNP levels, compared with discharge, were significantly higher at *p* < 0.001, *p* < 0.001, and *p* < 0.001 respectively. The 3-year Kaplan–Meier analysis, based on tertiles, revealed, for sαKlotho levels from Tertile 1 on admission and at discharge, a 2-fold higher rate of all-cause mortality or rehospitalization for HF compared with Tertile 3 (*p* = 0.006 and *p* = 0.028, respectively). One-third of patients showed an increase in FGF-23 and sαKlotho levels during hospitalization. Patients with the highest percentage increase in the levels of both biomarkers had an elevated risk of all-cause morality or hospitalization for HF (hazard ratio, 2.75; confidence interval, 1.19–6.35; *p* = 0.02). **Conclusions:** sαKlotho and FGF-23 levels are elevated during an episode of AHF. Low sαKlotho levels are associated with an increased risk of all-cause mortality or rehospitalization for HF. Increases in sαKlotho and FGF-23 values during hospitalization identify patients with poor prognosis.

## 1. Introduction

Acute heart failure (AHF) is a life-threatening stage with the rapid or progressive development of subjective and/or physical symptoms of heart failure (HF). It is characterized by accelerated myocardial necrosis and pathological remodeling [1,2,3]. Numerous clinical parameters and biomarkers serve as independent predictors of in-hospital complications and poor prognosis in AHF [4]. Extracardiac factors significantly contribute to the progression of HF and activate mechanisms of mutual damage [5]. In recent years, there have been numerous reports on the effects of hormonal mechanisms, mineral metabolism, and bone metabolism on premature atherosclerosis, pathological cardiovascular remodeling, and HF symptoms [6,7,8,9]. Two important biomarkers involved in these processes are fibroblast growth factor 23 (FGF-23) and soluble α-Klotho protein (sα-Klotho).

FGF-23 belongs to the phosphatonin family and is primarily produced in the bones. Together with calcitriol and parathyroid hormone (PTH), it plays a crucial role in maintaining phosphate homeostasis. FGF-23 reduces phosphatemia by inhibiting phosphate reabsorption in the proximal tubule of the kidneys. In addition, it lowers the levels of PTH and active vitamin D [10]. Recent data indicate that FGF-23 is also synthesized in various organs in response to stress. Cardiac FGF-23 is involved in interactions among cardiomyocytes, fibroblasts, coronary endothelial cells, and macrophages, leading to myocardial fibrosis and hypertrophy. Thus, it appears that pathological myocardial remodeling induced by FGF-23 occurs not only through endocrine pathways but also via paracrine and autocrine pathways [11]. The production of FGF-23 is stimulated mainly by dietary phosphate, acute or chronic renal dysfunction, calcitriol, PTH, calcium ions, iron deficiency, and inflammation. At the cellular level, the action of FGF-23 is mediated by the fibroblast growth factor receptor (FGFR) and the transmembrane Klotho protein [12]. Recent publications showed that increased levels of FGF-23 are associated with the highest cardiovascular risk [13,14,15]. Data indicate that FGF-23 has been recognized as a prognostic marker in chronic HF and a potent tool for the identification of right ventricle (RV) dysfunction in populations with a reduced left ventricle ejection fraction [16,17,18,19].

The Klotho protein, discovered by Kuro-o et al. and originally identified as an anti-aging protein, is a multifaceted molecule with pleiotropic properties [20]. It regulates calcium–phosphate metabolism, inflammatory processes, and glucose metabolism. In addition, it reduces oxidative stress and apoptosis and exhibits anti-fibrotic properties. Klotho was also reported to inhibit cancer growth [12,21,22,23], and its deficiency is associated with an increased number of episodes of arrhythmia [24,25]. The α-Klotho protein, produced by the Klotho gene is primarily present as isotype α and occurs in two forms: the transmembrane form, which acts as a co-receptor for FGF-23, and the secreted form. Membrane-bound α-Klotho is subjected to shedding by proteases to produce the so-called shed α-Klotho. Both the secreted and shed forms together constitute soluble α-Klotho (sαKlotho), which can be found in the plasma, cerebrospinal fluid, and urine [26,27].

In our previous study, we demonstrated elevated levels of sαKlotho during an episode of AHF, as well as its potential protective function, depending on its kinetics [28]. However, FGF-23 was not assessed in that study. Recently, there have been numerous reports on the effects of both biomarkers and their interactions in inflammatory processes and fibrosis in populations with stable coronary artery disease, chronic kidney disease, or respiratory disease [29,30,31]. In addition, extensive research has been conducted on the potential therapeutic use of sαKlotho in various civilization diseases [23,31].

The present study aimed to investigate the associations between FGF-23 and sαKlotho levels and the risk of all-cause mortality or hospitalization for HF in a population of patients with acute stage of HF.

## 2. Materials and Methods

### 2.1. Study Design

We analyzed 104 consecutive patients treated for AHF in the Intensive Cardiac Care Unit (ICCU) between June 2019 and January 2021. All patients received treatment in accordance with the current guidelines of the European Society of Cardiology and at the discretion of their attending physician [1]. Inclusion criteria were a diagnosis of AHF within 24 h of admission, and hospitalization that involved treatment with at least one of the following: diuretics in intravenous form, catecholamines, or mechanical circulatory support. Persons under 18 years and those with cardiogenic shock, cancers, autoimmune disorders, or mental illness were not included.

### 2.2. Follow-up and Study End Points

The primary endpoint of the study was 3-year all-cause mortality or rehospitalization for HF. Since our patient population was small, most of the 3-year follow-up data came from the outpatient clinics of our University Hospital. In addition, we used data from general practitioners, as well as telephone contacts with patients and their families. Data on deaths came from hospital records or death certificates. Patients with more than one outcome were counted only once and were excluded from further analysis after the first episode. The study was conducted in agreement with the Declaration of Helsinki and was approved by the local Bioethics Committee.

### 2.3. Laboratory Tests

Blood samples (EDTA) were collected within the first 24 h of admission and then when the patient left hospital. Each sample was immediately centrifuged for 15 min at 4000 rpm to obtain plasma. Next, samples were frozen at −80° until analysis. Plasma sαKlotho concentrations were measured using the Human Soluble α-Klotho ELISA kit (Immuno-Biological Laboratories, Inc., Minneapolis, MN, USA), while FGF-23 levels were measured using a Human FGF23 ELISA (Biobryt Ltd., Cambridge, UK). To ensure the validity of the method, all measurements were performed in duplicate. Other biochemical parameters used in the analysis were determined in the hospital laboratory.

### 2.4. Statistical Analysis

Data were presented as medians and interquartile range (IQR), means ± standard deviation, or counts and percentages. The Shapiro–Wilk test was used to verify the type of distribution. Student’s *t*-test was used to compare differences between groups for normally distributed variables, and the Mann–Whitney U-test for nonnormally distributed variables. For comparing two parameters within a single group, the Wilcoxon test was used. The results on graphs were presented as forest plots. Cumulative survival for groups dichotomized by sαKlotho and FGF-23 tertiles were assessed using the Kaplan–Meier method. Multivariable Cox regression analyses were built including common, generally recognized variables in heart failure (age and sex), variables of kidney function (estimated glomerular filtration rate), heart failure severity (NT-pro BNP). Additionally, the proportional hazard assumption was checked using Schoenfeld scaled residuals. R software v. 4.2.2 was used for all statistical analyses (the R Foundation for Statistical Computing, Vienna, Austria); *p*-values < 0.05 were regarded as significant.

## 3. Results

### 3.1. Patient Population

In Table 1, we present all epidemiological and clinical data of the study population. The mean age of the patients was 65.8 ± 14.6 years. The majority were male (75%), and most had acute decompensation of HF (56.7%). Coronary etiology was found in 43.3%. The mean left ventricular ejection fraction was 31.4% ± 14. Multimorbidity was common in the study group, with the following conditions present: hypertension in 74.0%, diabetes in 40.4%, atrial fibrillation in 47.1%, chronic kidney disease in 28.8%, and chronic obstructive pulmonary disease in 13.5% of patients

### 3.2. Changes in the Levels of Biomarkers

The median FGF-23 levels on admission in the entire population were 1159 pg/mL (IQR, 293–4291 pg/mL), while the median sαKlotho levels were 671 pg/mL (IQR, 502–840 pg/mL) and the NT-pro BNP levels were 5362 ng/L (IQR, 3257–13,653 ng/L). At discharge, reductions in the levels were observed: FGF-23 decreased to 374 pg/mL (IQR, 94–2041 pg/mL), sαKlotho to 545 pg/mL (IQR, 403–740 pg/mL), and NT-pro BNP to 4371 ng/L (IQR, 920–5013 ng/L). The percentage reductions were as follows: Δ% FGF-23, −0.38% (IQR, −0.73% to 0.00%), *p* = 0.01; Δ% sαKlotho, −0.16% (IQR, −0.35% to −0.03%), *p* < 0.001; and Δ% NT-pro BNP, −0.67 (IQR, −0.82% to −0.28%), *p* < 0.001.

### 3.3. Associations Between Biomarker Levels and Study Endopoints

During the 3-year follow-up, there were 52 adverse events (50%), 40 deaths, and 30 rehospitalizations for HF. Seven patients died in the hospital. Patients who reached the composite endpoint were older (*p* < 0.001), predominantly male, had a lower body mass index, and had significantly higher NT-pro BNP levels, lower hemoglobin levels, and a lower estimated glomerular filtration rate (eGFR). They also had multimorbidity and multiple organ damage more often than the other group. These patients also had significantly lower sαKlotho levels at admission and a significantly lower reduction in the levels of this biomarker between admission and discharge compared with the group that did not reach the endpoint (Table 1). Moreover, there were no significant differences in FGF-23 levels on admission and at discharge, and no significant difference in the percentage reduction between admission and discharge between groups (Table 1).

**Figure 1:**
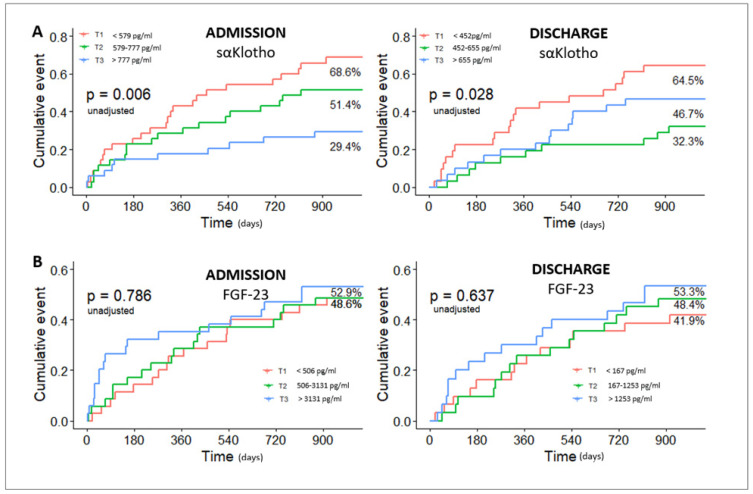
Kaplan–Meier curves and the relationships between sαKlotho (**A**) and FGF-23 (**B**) in tertiles and 3-year all-cause mortality or rehospitalizations for heart failure.

At 3 years, the analysis of sαKlotho levels by tertile showed a significantly higher risk of all-cause mortality and rehospitalization for HF for patients with sαKlotho levels below 579 pg/mL (Tertile 1) on admission and below 452 pg/mL (Tertile 1) at discharge (*p* = 0.06 and *p* = 0.028, respectively) (Figure 1A). No significant associations were observed for FGF-23 in the Kaplan–Meier analysis (*p* = 0.876 and *p* = 0.637, respectively) (Figure 1B).

Multivariable analysis showed that sαKlotho levels above Tertile 3 on admission were inversely and independently associated with the occurrence of the composite primary endpoint. In contrast, no association was found between FGF-23 levels and all-cause mortality or rehospitalization for HF (Table 2).

**Figure 2:**
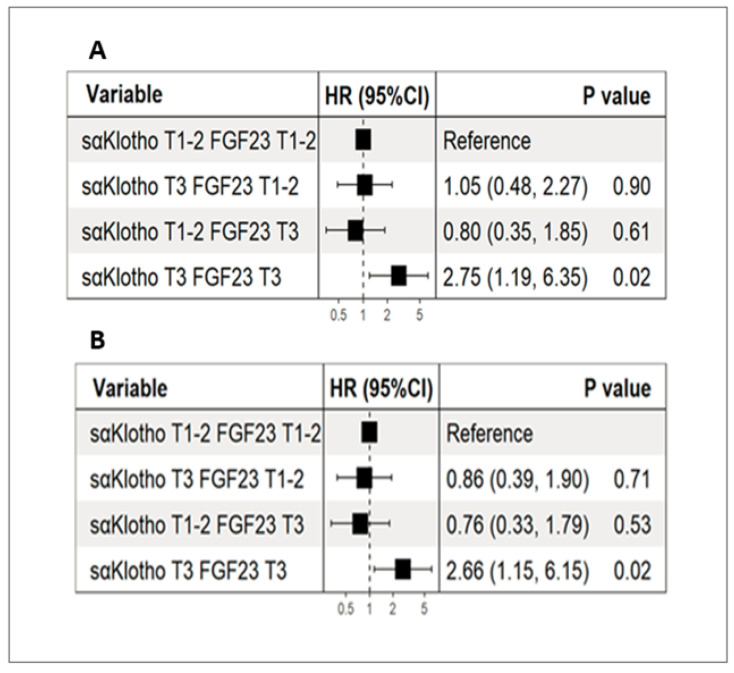
Forest plots showing risk of all-cause mortality or rehospitalization for heart failure stratified by percentage changes in sαKlotho and fibroblast growth factor 23 levels between admission and discharge: (**A**) unadjusted; (**B**) adjusted for age, male sex, NT-pro BNP, and eGFR. Abbreviations: CI, confidence interval; HR, hazard ratio; KT1-2, sαKlotho Tertiles 1-2; KT3, sαKlotho Tertile 3; FGF-23 T1-2, fibroblast growth factor 23 Tertiles 1-2; FGF23 T3, fibroblast growth factor 23 Tertile 3.

Although a reduction in biomarker levels during hospitalization was observed in the entire study population, the analysis by tertiles revealed that one-third of the patients (n = 32) showed no reduction in FGF-23 and sαKlotho levels, with some experiencing an increase between admission and discharge. For FGF-23, the percentage changes (Δ%) between admission and discharge were as follows: Tertile 1, −87 (−92 to −74); Tertile 2, −37 (−50 to −14); and Tertile 3, 27 (0.00 to 168), *p* = 0.01. For sαKlotho, the Δ% was Tertile 1, −40 (−47 to −35); Tertile 2, −15 (−21 to −10); and Tertile 3, 11 (−3 to 38), *p* < 0.001. At the 3-year observation, patients in Tertile 3, who presented the highest percentage increase in FGF-23 and sαKlotho levels between admission and discharge, had a 2-fold higher risk of all-cause mortality and rehospitalization for HF than patients in Tertiles 1 and 2, with a hazard ratio (HR) of 2.75 (95% CI, 1.19–6.35), *p* = 0.02 (Figure 2A). After adjusting for age, male sex, NT-pro BNP, and eGFR, the HR was 2.66 (95% CI, 1.15–6.15), *p* = 0.02 (Figure 2B).

**Figure 3:**
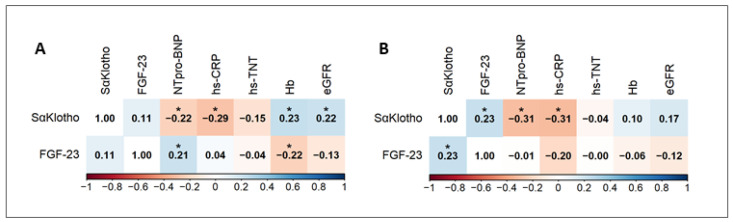
Correlation matrix of sαKlotho and FGF-23 at admission (**A**) and at discharge (**B**). Blue is a positive correlation and red is negative. Color intensity is proportional to the correlation coefficients. * *p* < 0.05; Hb, hemoglobin; CRP, C-reactive protein; eGFR, estimated glomerular filtration rate; NT-pro BNP, N-terminal pro-B-type natriuretic peptide.

On admission, sαKlotho levels showed a weak inverse correlation with NT-pro BNP (r = −0.22, *p* = 0.02) and high-sensitivity C-reactive protein (hs-CRP) (r = −0.29, *p* = 0.004) levels and a weak positive correlation with hemoglobin (r = 0.23, *p* = 0.02) and eGFR (r = 0.22, *p* = 0.03). On admission, FGF-23 levels showed a weak positive correlation with NT-pro BNP levels (r = 0.21, *p* = 0.03) and a weak inverse correlation with hemoglobin (r = −0.22, *p* = 0.02) (Figure 3A). At discharge, sαKlotho showed a weak inverse correlation with NT-pro BNP (r = −0.31, *p* = 0.04) and hs-CRP (r = −0.31, *p* = 0.003). FGF-23 levels showed a weak positive correlation only with sαKlotho levels (r = 0.23, *p* = 0.03) (Figure 3B).

## 4. Discussion

In our study, we demonstrated that in AHF, both sαKlotho and FGF23 levels are elevated at admission and exhibit dynamic changes. Fluctuations in the levels of both biomarkers were strongly associated with the occurrence of the primary composite endpoint of all-cause mortality or rehospitalization for HF. A lack of reduction in the levels of both biomarkers between admission and discharge identified patients with the poorest prognosis.

The pathophysiology of HF is influenced by numerous factors, including oxidative stress, neurohormonal activation, and inflammation [32]. These processes can occur at the tissue level or be present in the peripheral circulation. While initially adaptive, if they persist too long, they become detrimental, exacerbating HF and disrupting homeostasis, ultimately leading to degradation. Inflammation, oxidative stress, and neurohormonal factors were implicated in the progression of cardiac damage during both the acute phase of the disease and chronic stages following hospital discharge. Both FGF-23 and sαKlotho play a role in the aforementioned processes and are useful in predicting the risk of death and rehospitalization for subsequent episodes of AHF in the short and long term [33].

sαKlotho is a well-known molecule with pleiotropic properties [12]. Recent studies indicated that not only sαKlotho but also the entire sαKlotho/FGF-2 axis contributes to ongoing inflammation and the occurrence of fibrosis, leading to organ damage and cardiovascular disease [27,29,31]. In the PEACE study, patients with stable coronary artery disease, low sαKlotho, and high FGF-23 levels had a significantly higher risk of cardiovascular death or rehospitalization for HF [29].

Barnes et al. presented an interesting working hypothesis regarding the interaction between FGF-23 and sαKlotho. It is well established that the concentration of sαKlotho decreases due to aging and disease, which stimulates the production of transforming growth factor-β (TGF-β), a major cytokine involved in inflammatory processes and fibrosis. In response to elevated TGF-β levels, there is a compensatory production of FGF-23. This, in turn, stimulates an increase in sαKlotho levels to inhibit the abovementioned pathological processes. If the body cannot restore proper sαKlotho levels, inflammation and fibrosis persist, leading to organ damage and dysfunction [31]. In the absence of adequate sαKlotho levels, FGF-23 may induce cardiomyocyte hypertrophy, stimulate fibroblast activity leading to fibrosis, and increase the production of inflammatory cytokines in the liver. Additionally, by acting on the endothelium, it increases oxidative stress.

In our analysis by tertile, patients with the lowest Klotho values (Tertile 1), both on admission and at discharge, had significantly higher risk of all-cause mortality and rehospitalization for HF compared with the other patients (Figure 1A).

Our observations are consistent with other studies reporting an inverse association between sαKlotho levels and the incidence of cardiovascular disease [33].

In our previous study, we observed a significant increase in sαKlotho levels during an acute episode of HF compared with the control group, while a small reduction in sαKlotho levels during hospitalization was found to identify patients with poor long-term prognosis [28]. This finding may suggest a protective role for sαKlotho in AHF. Taneike et al., based on their population, also proposed sαKlotho as a new biomarker of response to treatment in AHF [34].

Reports regarding FGF-23 are less conclusive. Results from the multicenter TIME-CHF trial suggested a limited role for FGF-23 in predicting HF [35]. However, Plischke at al. showed the independent prognostic value of FGF-23 in a population of chronic HF patients [17]. An analysis of 980 HF patients presented by Koller et al. revealed a strong association between FGF-23 and the risk of mortality among patients with HF with reduced ejection fraction of left ventricle [18]. Vergaro et al. demonstrated that patients with AHF and high FGF-23 levels at discharge had a worse prognosis at 1-year follow-up. The authors additionally suggested that increase in FGF-23 during hospitalization could be associated with peripheral production in bone and kidney due to prolonged ischemia [14]. In our study, a 3-year Kaplan–Meier curve analysis indicated the worst prognosis in patients with FGF-23 levels in Tertile 3, but the results were not significant. In a multivariable analysis, male sex and age > 65 years were associated with the occurrence of the study endpoints. Only sαKlotho levels above Tertile 3 at admission were inversely and independently associated with all-cause mortality and rehospitalization for HF. There was no significant association between FGF-23 and outcomes at admission and at discharge.

Publications from recent years indicate emerging role of both biomarkers as a FGF-23/sαKlotho axis, in cardiovascular and metabolic prognostication [27,30,36,37]. Following this reasoning, the high levels of FGF-23 and sαKlotho in our population on admission should be regarded as compensatory mechanisms attempting to mitigate the pathological processes of inflammation and fibrosis. The present results strongly suggest that persons with an increased values of both biomarkers in Tertile 3 during hospitalization had worst prognosis during the 3-year observation and are also characterized by an inadequate response to treatment, despite therapy in accordance with the current guidelines. Thus, the sαKlotho/FGF-23 axis may probably serve as a predictor of response to treatment (Figure 2A,B).

Our study has several limitations. The data came from a single center, and the study group was relatively small. The database was based on consecutive patients, and the study protocol did not include any population selection. The etiology of AHF was heterogenous, which may have affected the results. Although we administered therapy according to the latest guidelines, a specific treatment protocol was not used. Clinical analyses based on relatively homogeneous, unique patient groups can strengthen group-specific results and reduce potential clinical factors that may confound the results. Therefore, our results should be considered as preliminary. Future studies on larger, diverse groups are clearly needed to reduce the potential biases.

## 5. Conclusions

Our study showed that sαKlotho and FGF-23 levels are upregulated during an acute episode of HF. A weak reduction in both biomarkers during hospital treatment due to AHF indicates a poor prognosis in the long-term follow-up. Higher levels of sαKlotho at admission are associated with better outcomes, suggesting a promising cardioprotective role for this biomarker in AHF.

## Figures and Tables

**Table 1:** Clinical characteristics of patients.

Variable	No Death or Rehospitalization for HF (n = 52)	Death or Rehospitalization for HF (n = 52)	*p*-Value
Demographic data			
Age, years	61.2 ± 17.5	70.5 ± 9.0	<0.001
Male, n (%)	35 (67.3)	43 (82.7)	0.07
New-onset AHF, n (%)	31 (59.6)	14 (26.9)	<0.001
Ischemic etiology, n (%)	16 (30.8)	29 (55.8)	0.01
BMI, kg/m^2^	31.7 ± 7.4	28.0 ± 5.3	0.004
Smoking, n (%)	18 (34.6)	24 (46.2)	0.23
Medical history			
Hypertension, n (%)	35 (67.3)	42 (80.8)	0.12
Myocardial infarction, n (%)	12 (23.1)	24 (46.2)	0.01
Diabetes, n (%)	17 (32.7)	25 (48.1)	0.11
CKD, n (%)	10 (19.2)	20 (38.5)	0.03
Atrial fibrillation, n (%)	20 (38.5)	29 (55.8)	0.07
ICD/CRTD/CRT, n (%)	4/4/1 (44.4/44.4/11.1)	12/5/0 (70.6/29.4/0)	0.23
Hospitalization data			
HR, bpm, admission	96.5 ± 25.0	92.4 ± 28.0	0.20
SBP, mmHg, admission	158.2 ± 39.5	136.0 ± 38.6	0.03
NYHA class IV, n (%), admission	38 (73.1)	40 (79.9)	0.83
Hospital stay, days	14 (9.0–22.0)	16 (9.0–27.3)	0.35
Norepinephrine, n (%)	4 (7.7)	6 (11.5)	0.51
Dobutamine, n (%)	1 (1.9)	8 (15.4)	0.02
Diuretics IV, n (%)	48 (92.3)	51 (98.1)	0.17
Levosimendan IV, n (%)	6 (11.5)	10 (19.2)	0.42
MCS—IABP, n (%)	2 (3.8)	1 (1.9)	0.56
β-blockers, n (%)	47 (90.4)	47 (90.4)	1.00
ARB, n (%)	5 (9.6)	4 (7.7)	0.73
ACEI, n (%)	40 (76.9)	31 (59.6)	0.06
MRA, n (%)	48 (92.3)	45 (86.5)	0.23
ARNI, n (%)	6 (11.5)	10 (19.2)	0.28
SGLT2 inhibitors, n (%)	3 (5.8)	4 (7.7)	0.70
Laboratory and echocardiographic parameters			
Hemoglobin, g/dL, admission	14.0 (11.8–15.8)	1.9 (10.6–14.3)	0.003
Creatinine, mg/dL, admission	1.1 (0.98–1.3)	1.3 (1.1–2.1)	0.02
eGFR, (mL/min/1.73 m^2^), admission	68.7 (51.2–78.5)	54.9 (34.5–75.0)	0.02
Bilirubin, mg/dL, admission	0.7 (0.38–0.95)	1.2 (0.62–1.69)	0.06
hsCRP, mg/dL, admission	10.4 (3.7–22.4)	10.1 (4.4–30.0)	0.50
hsCRP, mg/dL, discharge	5.6 (2.5–20.8)	8.4 (4.3–29.6)	0.05
hsCRP, delta%, admission–discharge	−44 (−75 to −6)	−10 (−65 to +38)	0.14
hsTnT, ng/L, admission	36.8 (24.1–126.0)	66.4 (32.7–148.7)	0.06
NT-pro BNP, ng/L, admission	4616.5 (3374.3–9636.3)	6457.5 (2913.5–17,603.3)	0.05
NT-pro BNP, ng/L, discharge	1452.0 (876.0–3026.0)	3331.5 (1183.0–9106.0)	0.03
NT-pro BNP, Δ%, admission–discharge	−72 (−85 to −50) *	−50 (−73 to −5) *	0.04
sαKlotho, pg/mL, admission	764.7 (598.8–874.2)	614.5 (463.8–742.1)	0.03
sαKlotho, pg/mL, discharge	551.8 (453.2–732.1)	514.6 (390.8–740.5)	0.30
sαKlotho, Δ%, admission–discharge	−22 (−35 to −8) *	−9 (−31 to +13) ^NS^	0.01
FGF-23, pg/mL, admission	1072.4 (253.1–3694.7)	1439.8 (368.8–5000.0)	0.46
FGF-23, pg/mL, discharge	357.9 (78.9–1289.5)	375.3 (152.4–2329.3)	0.34
FGF-23, Δ%, admission–discharge	−44 (−85 to 0) *	−14 (−69 to 0) **	0.22
LVEF, %, admission	32.3 ± 14.5	30.6 ± 12.8	0.54
LVEDd, mm, admission	59.5 ± 9.4	61.0 ± 9.8	0.49
TAPSE, mm, admission	16.3 ± 4.8	16.6 ± 5.3	0.82
IVC, mm, admission	21.9 ± 6.2	22.2 ± 8.3	0.79

Data are presented as the mean ± SD or median (IQR), unless indicated otherwise. * *p* < 0.001; ** *p* < 0.05; ^NS^, not significant. Abbreviations: ACEI, angiotensin-converting enzyme inhibitor; ADHF, acute decompensated heart failure; AHF, acute heart failure; ARB, angiotensin receptor blocker; ARNI, angiotensin receptor–neprilysin inhibitor; BMI, body mass index; CAD, coronary artery disease; CKD, chronic kidney disease; CRT, cardiac resynchronization therapy; CRTD, cardiac resynchronization therapy with defibrillator function; DCM, dilated cardiomyopathy; HR, heart rate; hsTnT, high-sensitivity troponin T; IABP, intra-aortic balloon pump; ICD, implantable cardioverter defibrillator; eGFR, estimated glomerular filtration rate; FGF-23, fibroblast growth factor 23; IV, intravenous; IVC, inferior vena cava; LVEF, left ventricular ejection fraction; LVEDd, left ventricular end-systolic dimension; MCS, mechanical circulatory support; MRA, mineralocorticoid receptor antagonist; NT-pro BNP, N-terminal pro-B-type natriuretic peptide; NYHA, New York Heart Association; SBP, systolic blood pressure; SGLT2, sodium-glucose cotransporter-2; sαKlotho, soluble αKlotho; TAPSE, tricuspid annular plane systolic excursion.

**Table 2:** Cox regression hazard analysis (all-cause mortality or HF rehospitalization) for sαKlotho (Tertile 3) and FGF-23 (Tertile 3) at admission and discharge.

	Admission		Discharge	
	HR (95% CI)	*p*-Value	HR (95%)	*p*-Value
Univariable				
sαKlotho (Tertile 3)	0.32 (0.15–0.67)	0.003	0.60 (0.30–1.18)	0.14
				
Multivariable				
sαKlotho (Tertile 3)	0.40 (0.16–0.77)	0.003	1.03 (0.47–2.24)	0.951
Age > 65 years	2.02 (1.07–3.81)	0.031	2.04 (1.02–4.05)	0.042
Male sex	2.65 (1.25–5.62)	0.010	2.45 (0.99–6.06)	0.050
NT-pro BNP	1.00 (1.00–1.00)	0.501	1.00 (1.00–1.00)	<0.001
eGFR	0.99 (0.97–1.00)	0.071	1.00 (0.98–1.02)	0.859
				
Univariable				
FGF-23 (Tertile 3)	1.24 (0.64–2.41)	0.502	1.42 (0.68–2.96)	0.302
				
Multivariable				
FGF-23 (Tertile 3)	1.00 (0.51–1.98)	0.996	0.66 (0.29–1.52)	0.331
Age > 65 years	2.10 (1.12–3.96)	0.021	2.14 (1.09–4.23)	0.032
Male sex	2.76 (1.28–5.95)	0.009	3.18 (1.25–8.12)	0.022
NT-pro BNP	1.00 (1.00–1.00)	<0.001	1.00 (1.00–1.00)	<0.001
eGFR	0.98 (0.97–1.00)	0.041	1.00 (0.98–1.02)	0.971

Abbreviations: CI, confidence interval; eGFR, estimated glomerular filtration rate; FGF-23, fibroblast growth factor 23; HR, hazard ratio; NT-pro BNP, N-terminal pro-B-type natriuretic peptide; sαKlotho, soluble αKlotho.

## Data Availability

The original contributions presented in this study are included in the article. Further inquiries can be directed to the corresponding author(s).
